# Management of patients with SARS-CoV-2 infections and of patients with chronic lung diseases during the COVID-19 pandemic (as of 9 May 2020): Statement of the Austrian Society of Pneumology (ASP)

**DOI:** 10.1007/s00508-020-01691-0

**Published:** 2020-06-12

**Authors:** Holger Flick, Britt-Madelaine Arns, Josef Bolitschek, Brigitte Bucher, Katharina Cima, Elisabeth Gingrich, Sabin Handzhiev, Maximilian Hochmair, Fritz Horak, Marco Idzko, Peter Jaksch, Gabor Kovacs, Roland Kropfmüller, Bernd Lamprecht, Judith Löffler-Ragg, Michael Meilinger, Horst Olschewski, Andreas Pfleger, Bernhard Puchner, Christoph Puelacher, Christian Prior, Patricia Rodriguez, Helmut Salzer, Peter Schenk, Otmar Schindler, Ingrid Stelzmüller, Volker Strenger, Helmut Täubl, Matthias Urban, Marlies Wagner, Franz Wimberger, Angela Zacharasiewicz, Ralf Harun Zwick, Ernst Eber

**Affiliations:** 1 Division of Pulmonology, Department of Internal Medicine, Medical University of Graz, Graz, Austria; 2 Department of Internal Medicine I, Hanusch Krankenhaus, Vienna, Austria; 3 Elisabethinen Hospital Linz, Linz, Austria; 4 Department of Pulmonology, Tirol Kliniken, Hospital Hochzirl-Natters, Natters, Austria; 5 Private Practice in Pulmonology, Vienna, Austria; 6 Department of Pulmonology, University Hospital Krems, Krems, Austria; 7 Respiratory Oncology Unit, Karl Landsteiner Institute of Lung Research and Pulmonary Oncology, Department of Internal and Respiratory Medicine, Krankenhaus Nord—Klinik Floridsdorf, Vienna, Austria; 8 Allergy Center Vienna West, Vienna, Austria; 9 Division of Pulmonology, Department of Internal Medicine II, Medical University of Vienna, Vienna, Austria; 10 Division of Thoracic Surgery, Department of Surgery, Medical University of Vienna, Vienna, Austria; 11 Ludwig Boltzmann Institute for Lung Vascular Research, Graz, Austria; 12 Department of Pulmonology, Kepler University Hospital, Medical Faculty, Johannes Kepler University, Linz, Austria; 13 Department of Internal Medicine II (Infectious Diseases, Pneumology, Rheumatology), Medical University of Innsbruck, Innsbruck, Austria; 14 Department of Internal and Respiratory Medicine, Krankenhaus Nord—Klinik Floridsdorf, Vienna, Austria; 15 Division of Paediatric Pulmonology and Allergology, Department of Paediatrics and Adolescent Medicine, Medical University of Graz, Auenbruggerplatz 34/2, 8036 Graz, Austria; 16 Division of Pulmonology, Reha Zentrum Münster, Münster, Austria; 17 Interdisciplinary Outpatient Sleep Laboratory, Telfs, Austria; 18 Private Practice in Pulmonology, Innsbruck, Austria; 19 Department of Pulmonology, Landesklinikum Hochegg, Grimmenstein, Austria; 20 Department of Internal, Respiratory and Critical Care Medicine, State Hospital II, Location Enzenbach, Gratwein-Straßengel, Austria; 21 Private Practice in Pulmonology, Salzburg, Austria; 22 Department of Paediatrics, Teaching Hospital of the Medical University of Vienna, Wilhelminen Hospital, Vienna, Austria; 23 Therme Wien Med, Vienna, Austria

**Keywords:** SARS-CoV‑2, COVID-19, Community acquired pneumonia, ARDS, Chronic lung disease

## Abstract

The coronavirus disease 2019 (COVID-19) pandemic is currently a challenge worldwide. In Austria, a crisis within the healthcare system has so far been prevented. The treatment of patients with community-acquired pneumonia (CAP), including SARS-CoV‑2 infections, should continue to be based on evidence-based CAP guidelines during the pandemic; however, COVID-19 specific adjustments are useful. The treatment of patients with chronic lung diseases has to be adapted during the pandemic but must still be guaranteed.

## Introduction

The Austrian healthcare system is currently confronted with the challenge of the coronavirus disease 2019 (COVID-19) pandemic. Since March 2020, incisive adaptations to the thus far well-established medical care structures and procedures have been rapidly implemented in order to be prepared for a high number of acutely and severely ill patients suffering from COVID-19. Simultaneously, the speed of the spread of SARS-CoV‑2 in Austria could be effectively reduced by radical, preventive social measures and a critical overburdening of the medical care centres has so far been successfully prevented.

In the current situation, there are three goals for pneumologists:

1. Optimal medical care for severely ill patients suffering from COVID-19 in order to achieve the lowest possible SARS-CoV‑2 mortality rate.
2. Guarantee of an unchanged best possible medical acute care of patients with other severe pulmonary diseases (infections, asthma, chronic obstructive pulmonary disease (COPD), interstitial lung disease (ILD) or cystic fibrosis (CF) exacerbations, pulmonary embolism, probable malignant pulmonary lesions, etc).
3. Continuation of important medical treatment of people with underlying severe chronic diseases (lung cancer, asthma, COPD, pulmonary hypertension, ILD, CF, status post lung transplantation, sleep associated breathing disorders, etc.). These patients require special attention, because they could be further threatened by a SARS-CoV‑2 infection.

In order to attain all three of the aforementioned goals, as far as the current resources (which are limited due to the pandemic) allow, alignment of medical activities with existing evidence-based and well-implemented guidelines and their adaptation to the currently difficult situation, as might be required in individual cases, should be continued. Especially with respect to chronic diseases, acting with good judgement and open communication with patients and relatives are required to find feasible solutions.

## Management of patients with SARS-CoV-2 infections

### The current epidemiological situation

#### General facts on COVID-19

Since January 2020, the COVID-19 pandemic has spread rapidly worldwide. According to the World Health Organization (WHO) up to now 3,759,967 COVID-19 cases have been confirmed worldwide and 259,474 patients have already died [1].

Epidemiological information on and study results from COVID-19 must still be interpreted with caution. They are subject to powerful dynamics and multifactorial influences, display a variable data quality and due to differences in healthcare structures and epidemiological features allow only limited international comparisons. Therefore, as is common practice in antibiotic stewardship, national and regional data should be systematically gathered and regularly analyzed. This is the only way in which the current local situation can be adequately assessed.

Like influenza, COVID-19 is a viral infectious disease with a variable course (from asymptomatic to mild to severe to fatal). In Europe, most of the people positively tested show mild symptoms. Conversely, more than 80% of the hospitalized patients suffered from fever, cough and respiratory distress (Table 1; [2, 3]). In particular, older and comorbid patients are severely affected and present with severe community acquired pneumonia (CAP) with resulting hypoxia. In addition, possible COVID-19 specific phenomena are described, such as a reduced sensation of dyspnea, whereby a respiratory deterioration may not be subjectively perceived for a long time, a lack of increase of the respiratory rate despite severe oxygenation disturbance, and a temporary loss of smell and taste.

**Table 1:** Symptoms of a SARS-CoV‑2 infection [2, 3]

Symptoms	Positively tested people (including mild cases)	Hospitalized COVID-19 patients
Fever/chills	49%	85%
Cough	24%	86%
Shortness of breath	–	80%
Myalgia	–	34%
Diarrhea	2%	27%
Nausea/vomiting	–	24%
Sore throat	12%	18%
Headache	–	16%
Nasal congestion, rhinorrhea	4%	16%
Chest pain	–	15%
Abdominal pain	–	8%
Fatigue	8%	–
Aching	7%	–

Due to the infectiousness of the pathogen, hospital-associated SARS-CoV‑2 pneumonia can also be expected in the future.

According to the European Centre for Disease Prevention and Control (ECDC), severe COVID-19 courses (need for hospitalization) have so far been observed in Europe in 28% of all cases; however, due to undetected mild courses, a high number of unreported cases and a higher rate of mild courses can be assumed.

An average of 16% of hospitalized patients suffered from a very serious illness course (need for intensive care or respiratory support) and COVID-19 hospital mortality in Europe is currently at 14% [2].

There are relevant differences in Europe with respect to COVID-19 deaths per 100,000 inhabitants. With a comparable COVID-19 incidence (175–250 cases/100,000 inhabitants), 7–9 deaths/100,000 have been registered in Austria, Denmark, Germany, and Liechtenstein and 31–39 deaths/100,000 in France, Sweden, and the Netherlands [4]. In Europe, the highest burden of COVID-19 is currently reported from Belgium (455 cases/100,000 and 75 deaths/100,000 inhabitants). In line with these figures, the European monitoring of excess mortality for public health action (EuroMOMO) network has recorded an exceptionally high pandemic-associated excess mortality rate in certain European countries (UK, France, Spain, Belgium, the Netherlands, Italy, and Switzerland), but a significantly lower one in Austria and other countries, such as Denmark, Germany, Greece, Norway, and Ireland [5].

In Austria, 15,735 persons have so far been tested positive for SARS-CoV‑2 and 615 (3.9%) have died from or with COVID-19. At present, 230 COVID-19 patients are hospitalized (peak at the beginning of March with 1010 hospitalized patients) and 79 are being treated in intensive care units (peak at the beginning of March with 267 ICU patients) (24–34% more than the European average). Thus, at the beginning of March 26% (currently only 8%) of all available intensive care beds in Austria were occupied by COVID-19 patients [6]. Primary data on the number of patients previously treated in hospitals or intensive care units and the mortality rates are currently unavailable in Austria.

#### Hospitalization and mortality risk for COVID-19 and community-acquired pneumonia due to other pathogens

In order to realistically classify the current COVID-19 data, they must also be compared with the incidence and course of other severe respiratory infections as they occurred before the COVID-19 pandemic. In principle, pathogen-induced CAP which requires hospitalization (hCAP) is frequent. With an incidence of 296 hCAP per 100,000 inhabitants, an estimated 26,222 patients with hCAP are treated in Austria every year and 2185 patients every month [7]. With an average hospital mortality rate of 13% (Table 2) Austria has 3409 (39/100,000) hCAP deaths per year and 284 hCAP deaths per month whereas COVID-19 caused 491 deaths per month during the peak phase of the pandemic (27 March–27 April 2020). It can therefore be assumed that in Austria the pandemic caused at least a transient doubling of hCAP deaths/100,000 inhabitants.

**Table 2:** Hospital and ICU mortality rates for COVID-19 worldwide as compared to other CAP-associated pathogens from solely European and North American studies

	Hospital mortality	ICU mortality
**CAP in general** [7, 15–19]	12.9–14.1%	17.0–29.5%
*S. pneumoniae* [18, 20, 21]	8.0–12.0%	17.5–26.0%
*L. pneumonia* [22–25]	3.9–18.5%	21.6%
**Viral CAP in general** [26, 27]	14.8%	22.0%
**Influenza A/B** [10, 19, 28–31]	12.6%	17.1–41.2%
**COVID-19**		
China (Wuhan)^a^ [32–36]	10.7–21.9%	61.5%
USA (New York)^b^ [37]	21.0%	78.0%
Europe (ECDC) [2]	14%	–
United Kingdom^a^ [26]	–	34.8–46.8%
Spain^a^ [38]	–	29.2%
Italy (Lombardy)^a^ [39]	–	25.6%

^a^ COVID-19 pandemic epicenters

^b^Epicenter New York: on 23 April 2020 approx. tenfold more SARS-CoV‑2 infected people/100,000 inhabitants and 20-fold more COVID-19 deaths/100,000 inhabitants than in Austria at the same time [40]

*CAP* community acquired pneumonia, *COVID-19* coronavirus disease 2019, *ECDC* European Centre for Disease Prevention and Control, *ICU* intensive care unit

Influenza must be considered separately as the influenza case fatality rate is only partly caused by influenza pneumonia but 400,000 influenza-associated deaths are annually expected worldwide [8, 9].

The incidence of inpatient influenza cases in Europe ranges between 12–95/100,000 depending on the season of the year and the effective vaccination coverage rate of the population, and for children in Austria between 2002 and 2018 was 50/100,000 [10–13]. If this incidence is applied to Austria, assuming an ICU rate of 7% and a hospital mortality rate of 4%, during each influenza season there will be 1152–8416 inpatients, 81–589 cases requiring ICU, and 46–337 inpatient deaths in Austria. For the period from December to April (influenza season), for Austria this means that there are 288–2104 inpatients and 20–147 influenza cases requiring ICU per month. Due to a very low influenza vaccination rate as compared to other European countries, higher rather than lower rates can be expected for Austria. This assumption is supported by calculations of the Agentur für Gesundheit und Ernährungssicherheit (AGES), which based on the statistical model FluMOMO, supposes an average of 2326 influenza deaths per year in the last 4 years and thus 582 influenza deaths per month during the influenza season (COVID-19: currently approximately 450 deaths per month, as of 19 April 2020) [14]. Accordingly, the annual wave of influenza in Austria is very likely to lead to a burden on the healthcare system comparable to that of the current COVID-19 pandemic. Therefore, systematic recording like that currently established for COVID-19 should also be introduced in Austria with respect to influenza-associated deaths amongst hospitalized patients.

To sum up, it can be assumed that SARS-CoV‑2 can be listed as another relevant CAP pathogen that for an unforeseeable period of time will significantly increase the incidence of CAP, especially amongst older people, and similarly to influenza infections involves significant logistical and hospital hygiene and infection prevention efforts. Due to the governmental preventive measures both inside and outside the healthcare system, the COVID-19 pandemic in Austria has so far been successfully contained but a further increase in COVID-19 cases is possible following the easing of the lockdown restrictions. As far as can be currently assessed, the hospitalization rate for SARS-CoV‑2 CAP seems to be higher than that for CAP due to other pathogens, and depending on the functionality of the healthcare system hospital mortality would appear to be comparable to that of other pathogen-induced CAP (Table 2).

The CAP mortality risk is determined by the extent of immediate lung parenchyma damage, secondary infections/complications, age and pre-existing comorbidities, and the quality of the available medical care. The significance of typical cardiopulmonary, renal and metabolic comorbidities for the course of CAP is well-known from influenza, pneumococcal and legionella infections, and plays a decisive role in SARS-CoV‑2 CAP to the same extent. Consequently, as is the case with other CAP pathogens, the risks of hospitalization and mortality of SARS-CoV‑2 CAP increase significantly from the age of 60 years and with the number of concomitant diseases (Table 3; [7, 10, 15, 29, 41]).

**Table 3:** Comparison of comorbidities of patients who died from COVID-19 or other pneumonia pathogens (pneumococcus, influenza, etc.) [41–44]

Comorbidities of deceased patients	COVID-19 (%)	Other CAP pathogens (%)
Arterial hypertension	40–75	54
Diabetes	20–31	31
Heart diseases	23–49	38
Neurologic disorders	13	16–19
Carcinomas	2–18	28
Chronic renal insufficiency	23	13–27
Chronic lung diseases	8–19	6–24
Dementia	18	28

*CAP* community acquired pneumonia, *COVID-19* coronavirus disease 2019

Furthermore, the COVID-19 pandemic has clearly demonstrated that the mortality rate of an acute infection is always determined by social and structural factors (e.g. timely public health interventions to slow the spread of a pandemic infection, prompt and flexible structural adjustments to the healthcare system, the number of immediately available intensive care or mechanical ventilation beds, capacity for isolation and protection in the outpatient and inpatient area, short-term and effective medical staff training).

In some countries and regions there were acute supply emergencies and therefore it can be assumed that in these critical and partly catastrophic medical situations, not all acutely and severely ill patients could be provided with the required timely and adequate medical care. For example, the mortality rate in the primarily unprepared epicenter (Wuhan city in Hubei province) was initially 12% and later in the other Chinese provinces only about 1% [45]. This is substantiated by excess mortality rates recorded by EuroMOMO in some countries that were severely affected by the pandemic.

#### SARS-CoV-2 in children

In an analysis of the first approximately 45,000 laboratory-confirmed COVID-19 cases in China, children <10 years of age represented only 0.9% (416 children) and children between 10 and 19 years of age only 1.2% (549 children) of the cases [46]. Until now, neonatal COVID-19 cases have been extremely rare [47]; however, the number of children, who are actually infected, but have not been tested due to missing or mild symptoms, still remains unclear. Close contact with a SARS-CoV‑2 infected person in the family environment seems to be the most frequent transmission route [48].

As compared to adults, children and adolescents are much less likely to fall ill from SARS-CoV‑2 and have often shown only mild clinical symptoms. Only one quarter developed temperatures between 38 and 39 °C, and only 10% temperatures >39.0 °C. Coughing and tachypnea are described in about 30–50% and pharyngitis (5–45%), rhinitis (10–30%), diarrhea (10–30%) and vomiting (6%) are significantly less frequent [48–51]. Similarly to adults, laboratory tests showed an increase in C-reactive protein (CRP) (moderate), transaminases, lactate dehydrogenase, D‑dimer and creatine kinase, as well as leukopenia (primarily lymphopenia) [51].

Due to the less specific symptoms in children, it is difficult to make a reliable clinical diagnosis. Accordingly, especially in pediatric patients it is important to test extensively for SARS-CoV‑2 and to implement appropriate protective measures for medical personnel.

Severe courses of respiratory insufficiency, or the need for intensive care constitute the exception [47]. Severe COVID-19 infections have been repeatedly suspected in infants; however, these were mostly only suspected cases (without SARS-CoV‑2 testing). The authors assume that other viruses (especially respiratory syncytial virus [RSV]) might have caused a considerable percentage of the severe courses of the infection [52]. Only a few pediatric COVID-19 deaths have been reported in the literature so far [46, 47, 53].

Due to the often milder disease course in children, it has been discussed whether oligosymptomatic and asymptomatic children could play an essential role in the transmission, without this hypothesis ever having been confirmed scientifically [52]. On the contrary, a recent study from Iceland showed that when screening asymptomatic individuals, the proportion of virus excretion is threefold higher in 40–50 year-olds (approx. 1.5%) than in children/young people between the ages of 10 and 20 years (approx. 0.5%). In a group of more than 800 children under 10 years of age, not a single child was tested positive [54].

The SARS-CoV‑2 infections in children with risk factors and underlying diseases (chronic respiratory diseases such as cystic fibrosis, severe asthma, bronchopulmonary dysplasia as well as cardiac diseases, primary and secondary immunodeficiencies, underlying malignant diseases, malnutrition, etc.) are rarely reported in pediatric analyses [46, 52]. Whether or not it can be derived that these children are less at risk than adults with risk factors, or whether children from risk groups have more effectively been protected against infection, remains unclear.

#### Epidemiological outlook

As soon as the governmental pandemic prevention measures are eased, the Austrian healthcare system must be further prepared for more than a renewed increase in the number of COVID-19 cases. All other respiratory infections (e.g. influenza, RSV, *Pneumococcus, Mycoplasma* and *Bordetella* infections), the spread of which as in the case of SARS-CoV‑2 was concomitantly suppressed by the pandemic prevention measures, will also increase again.

**Fig. 1:**
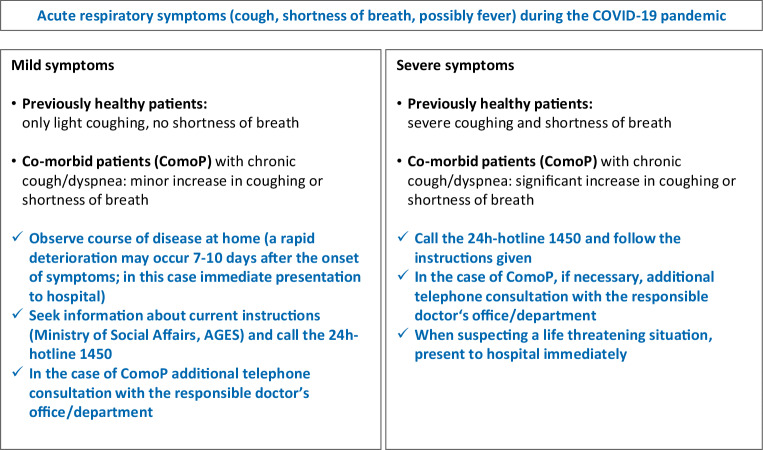
Guidance for patients regarding the severity of a possible SARS-CoV‑2 infection

Within this context, the increased public awareness of potentially threatening infectious diseases created by the COVID-19 pandemic is to be welcomed. As a next step, targeted reasonable, individual and social preventive measures have to be developed and supported. For example, these could not only include the individual willingness for protective vaccination against influenza and other relevant pathogens but also a deeper understanding among the population of how to autonomously differentiate between harmless infections that should be cured at home and serious acute illnesses that must be treated by a general practitioner or in hospital (Fig. 1).

### Management of SARS-CoV-2 pneumonia

#### Basic management of SARS-CoV-2 CAP

Serious SARS-CoV‑2 pneumonia is a severe viral CAP (svCAP), the clinical presentation of which (acute onset, bilateral pneumonia, progressive respiratory failure, high risk of mortality) is comparable to that of severe influenza CAP (Table 2). In the current pandemic situation, the guarantee of sufficient medical care for such severe medical conditions is of crucial importance. Due to the frequency of svCAP (especially during the annual influenza season), the medical centers in Austria are familiar with the clinical management of svCAP.

**Fig. 2:**
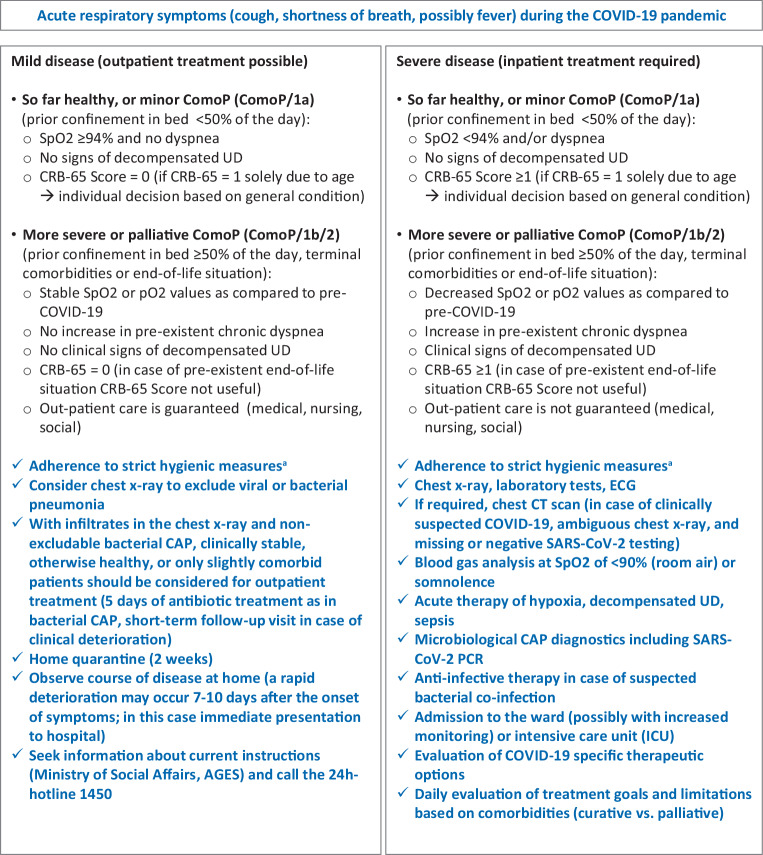
Guidance for physicians regarding the degree of severity of a probable SARS-CoV‑2 infection (adapted from [55, pp. 151–200]). ^a^Robert Koch Institute guidelines on hygienic measures within the framework of the care and nursing of patients with a SARS-CoV‑2 infection: https://www.rki.de/DE/Content/InfAZ/N/Neuartiges_Coronavirus/Hygiene.html. *CAP* community-acquired pneumonia, *CRB* confusion, respiratory rate, blood pressure, *CRB-65 score* RR ≥30/min, diastolic blood pressure ≤60 mm Hg or systolic blood pressure <90 mm Hg, disorientation, age ≥65 years, *UD* underlying disease, *ComoP/1b* comorbid patients with prior confinement in bed ≥50% of the day, *ComoP/2* comorbid patients with previously infaust prognosis, independent of a probable or confirmed SARS-CoV‑2 infection

**Fig. 3:**
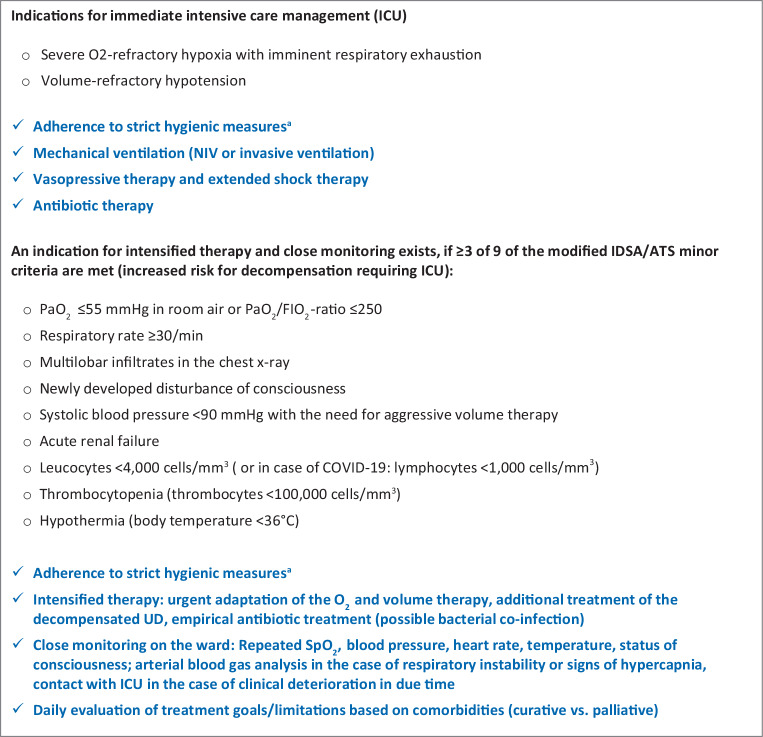
Guidance for the identification of critically ill CAP patients during the COVID-19 pandemic (CAP as an emergency) (adapted from [55, pp. 151–200]). ^a^Robert Koch Institute guidelines on hygienic measures within the framework of the treatment and care of patients with a SARS-CoV‑2 infection: https://www.rki.de/DE/Content/InfAZ/N/Neuartiges_Coronavirus/Hygiene.html. *CAP* community-acquired pneumonia, *UD* underlying disease, *IDSA/ATS* Infectious Diseases Society of America/American Thoracic Society, *NIV* non-invasive ventilation, *ICU* intensive care unit

As the functionality of the Austrian healthcare system was not significantly impaired during the current COVID-19 pandemic, the key points of current evidence-based guidelines for the treatment of CAP should also be applied to SARS-CoV‑2 CAP and serve as general orientation (Figs. 1, 2 and 3):

- Early diagnosis of CAP, possibly simultaneously decompensated underlying diseases and the recognition of life-threatening situations
- Start of CAP therapy without delay (including the treatment of respiratory insufficiency, hemodynamic instability, decompensated underlying diseases and, if indicated, anti-infective therapy)
- Triage according to the clinical findings (outpatient vs. inpatient vs. intensive care treatment)
- Definition of appropriate treatment goals and avoidance of futile treatment in palliative patients already suffering from severe underlying diseases (see below)
- From the outset, consequent adherence to strict hygiene measures for personal protection and the avoidance of nosocomial infections
- Prevention of new infections

For details regarding guideline-based CAP diagnosis and therapy refer to current guidelines [55].

#### Diagnostics

The key signs and symptoms of almost all respiratory tract infections are cough, possibly fever and dyspnea. This applies to any kind of bacterial or viral acute bronchitis, COPD exacerbation or pneumonia including COVID-19. Although COVID-19 is currently being diagnosed in hospitals to a larger extent, other acute cardiorespiratory diseases and infections are also present and will likewise increase again after the liberalization of the social pandemic measures. Therefore, CAP diagnostics that are exclusively focused on COVID-19 is unreasonable. In Austria CAP diagnostics should generally continue to follow the current recommendations from the German-Austrian-Swiss CAP guideline for adults from 2016 and for children and adolescents from 2017 [55, 56].

Nevertheless, within the context of the COVID-19 pandemic, diagnostic amendments for the early detection of a SARS-CoV‑2 infection in routine diagnostics are necessary (Figs. 1, 2 and 3):

- Outpatients who are not seriously ill should contact the established contact points by telephone and seek information and advice on the current procedure (Fig. 1).
- In patients with the clinical presentation of a possible respiratory tract infection without a clear etiological attribution, a SARS-CoV‑2 PCR should be performed in emergency rooms, or in hospitals if it is of therapeutic or hygienic relevance (Figs. 2 and 3).
- A chest CT scan without contrast agent should be performed in patients in the emergency department or already hospitalized if a lower respiratory tract infection is suspected
  **AND**
  the chest x‑ray is unremarkable (or difficult to interpret)
  **AND**
  the rapid diagnostic tests for common infections (Influenza/RSV/SARS-CoV‑2 PCR, *Pneumococcus*/*Legionella* urine antigen test) are negative
  **AND**
  typical laboratory values for COVID-19 (leucocytes <10.0 × 10^9^/L, neutrophils <7.0 × 10^9^/L, lymphocytes <1.0 × 10^9^/L, CRP only moderately elevated (10–130 mg/L), procalcitonin <1.0 ng/mL [34, 37]) are present.

With typical COVID-19 CT findings, but a negative SARS-CoV‑2 PCR, the patient should first be classified as a suspected COVID-19, and other differential diagnoses proactively evaluated and the SARS-CoV‑2 PCR repeated.

A positive SARS-CoV‑2 PCR confirms the diagnosis of COVID-19. The sensitivity of a virus-specific PCR is dependent on multiple factors, such as the time of testing (at the start of infection versus a later time point), the sample material (oropharyngeal swab versus nasopharyngeal swab versus sputum or bronchial lavage), the sample quality and the applied test procedure (type of assay). Therefore, a negative PCR result does not exclude COVID-19 if the clinical presentation and the CT findings are typical. The SARS-CoV‑2 PCR from sputum samples or bronchial lavage fluids are in general more sensitive than those from nasopharyngeal smears [57]; however, for reasons of hygiene neither sputum induction nor diagnostic bronchoscopy should be solely performed for confirming COVID-19. In intubated patients with an initially negative PCR from the upper respiratory tract, further PCR testing in a lower respiratory tract specimen (e.g. tracheal secretions via closed suction system) is recommended. This increases the diagnostic sensitivity and reduces the false negative test rate [58, 59].

A chest x‑ray is neither sufficiently sensitive nor precise enough for the diagnosis of SARS-CoV‑2 CAP; however, if the clinical signs and symptoms are specific and the PCR result is positive, x‑ray findings typical for COVID-19 (bilateral mostly ground glass-like peripheral and basal consolidations) are sufficient.

In justified cases (as mentioned), severe cases, or for better differentiation of alternative diagnoses or complications, a chest CT scan is indicated [60]. Typical COVID-19 chest CT findings are bilateral, multifocal, peripheral/subpleural and dorsobasal ground glass opacities with or without consolidations. In the course of the disease, consolidation areas may increase and a crazy paving pattern may occur. Sensitivity, specificity, negative and positive predictive values of chest CT scans were described in a larger study as 97%, 25%, 65% and 83%, respectively [61]. Thus, SARS-CoV‑2 CAP can be detected sensitively by chest CT, but the radiological changes may also result from other infections or diseases, or complications.

#### Specific SARS-CoV-2 CAP therapy

In general, treatment of a SARS-CoV‑2 CAP, as of another bacterial or viral pneumonia, should follow relevant guidelines (*see* above). Currently, there is broad discussion about antiviral and anti-inflammatory treatment approaches that have yet to be sufficiently validated (remdesivir, chloroquine, hydroxychloroquine, tocilizumab, recombinant angiotensin converting enzyme 2 and others). They should therefore not be used as standard therapy in clinical routine. According to the WHO recommendations, their efficacy, safety and tolerability should first be tested in clinical trials, preferably randomized controlled trials (RCT) [62, 63]. Until results from RCTs are available, experimental therapies outside clinical trials must be extremely well justified and considered solely in selected individual cases (compassionate use). They should not be used uncritically, potentially harmful side effects must be considered and wherever possible, their application should be documented in registers [64].

On 28 March 2020, the U.S. Food and Drug Administration (FDA) issued an emergency use authorization for chloroquine/hydroxychloroquine, and on 1 May 2020 for remdesivir for the treatment of COVID-19 [65]. The FDA points out that only in vitro or anecdotal clinical data and case series on the efficacy of chloroquine and hydroxychloroquine are available and that these drugs should be further tested in RCTs. Nevertheless, the FDA has approved the use of chloroquine and hydroxychloroquine for hospitalized COVID-19 patients (body weight >50 kg) outside of studies. For remdesevir, the FDA decision was based on unpublished topline data from a randomized, double-blind, placebo-controlled trial (NCT04280705) and from another open-label trial (NCT04292899). At present, the European Medicines Agency (EMA) has not granted approval for chloroquine, hydroxychloroquine, remdesivir or any other specific SARS-CoV‑2 therapy or vaccination.

#### Systemic steroids

With a few exceptions, a large number of studies and meta-analyses showed no benefit and even an increased fatality rate for systemic steroids in svCAP or viral acute respiratory distress syndrome (vARDS) [66–68]. Accordingly, the routine use of systemic steroids for the treatment of svCAP/vARDS including COVID-19 is not recommended [62]; however, in exceptional circumstances, systemic steroids may be considered in cases of viral CAP:

- According to the septicemia guidelines, hydrocortisone is indicated for refractory shock with massive hemodynamic instability [69, 70].
- Severe COPD exacerbation: 0.5 mg prednisolone/kg/day for 5–7 days, then stop.
- Severe asthma exacerbation: 0.5 mg prednisolone/kg/day for a maximum of 7 days, then slowly tapering over a further 7 days.
- In the course of svCAP, systemic steroids may be considered in suspected individual cases of organizing pneumonia, postpneumonic interstitial pneumonia, hemophagocytic lymphohistiocytosis, or exacerbation of pre-existing pulmonary fibrosis.

#### Respiratory intensive care

Patients requiring intensive care and ventilation should be treated according to generally accepted national and international recommendations. Thus, for the usually predominant severe oxygenation disorder, an escalation from a ventilation mask with reservoir (non-rebreather mask) via high-flow nasal oxygenation (HFNO) to non-invasive ventilation (NIV) is recommended. In all international recommendations, special focus is placed on the protection of the practitioner, in particular during measures such as intubation, NIV, HFNO, bronchoscopy or nebulization [69, 71, 72]. Aerosol production is probably not significantly increased with oxygen therapy, HFNO, nebulization and NIV with non-vented systems, and a significantly increased risk for personnel is presently not assumed. In contrast, an increased risk for personnel has been shown for intubation, bronchoscopy, endotracheal aspiration and the use of vented systems, or in the absence of a virus filter in the expiratory part of ventilation systems. A recent COVID-19 position paper of the German Respiratory Society provided a good overview of aerosol production and the resultant risk for practitioners [73].

If available, HFNO and NIV of COVID-19 patients should be performed in negative pressure rooms. In clinical practice, however, the number of negative pressure rooms is limited in Austria, and HFNO and NIV are also acceptable in other facilities; however, personal protection measures must be strictly adhered to.

Since aerosol formation increases with augmented HFNO flow rate, the flow rate should be set as low as possible and an oronasal mask (FFP1 mask) should be applied to the patient’s face to reduce aerosol release. In general, intensified monitoring should be ensured in patients with very high or rapidly progressive oxygen demand because acute respiratory failure requiring immediate intubation must be identified without delay.

Irrespective of the type of ventilation, the use of a respirator with a double-hose system and bacteria/virus filter at the expiratory section of the breathing circuit is recommended. Ventilation with a single-hose system and vented systems should be avoided due to aerosol formation. Ventilators for home ventilation, including obstructive sleep apnoea syndrome (OSAS) therapy, should therefore not be used in the inpatient setting for SARS-CoV‑2 positive patients, but should be replaced by suitable ventilators, or an appropriate mask construction with a filter at the expiratory valve. Air humidifiers of home ventilators should not be used [74]. If only ventilators with a single-hose system and distal flow measurement are available, a filter must be installed at the patient side, with the resultant increase in airway resistance to be taken into account. If a continuous positive airway pressure (CPAP) helmet is used, a filter must be attached to the expiratory part.

For intubation, video laryngoscopy and rapid sequence induction with full relaxation are recommended to prevent aerosol formation, possible coughing of the patient and close proximity of the airway operator to the patient’s head. Nebulization should be avoided in favor of the use of metered dose inhalers.

According to the severity of the oxygenation impairment, intubation and invasive ventilation are often recommended for an oxygenation index (PaO2/FiO2) ≤200 [72]. Whether in such a case NIV is still feasible as an alternative has to be individually assessed for each patient. Depending on the underlying pulmonary disease and the clinical condition, with special regard to the load of the respiratory muscles, the cooperation of the patient, strict protective measures for the medical staff, and the user’s experience with NIV are particularly important. In the presence of ARDS and no improvement with NIV, intubation should not be delayed.

**Fig. 4:**
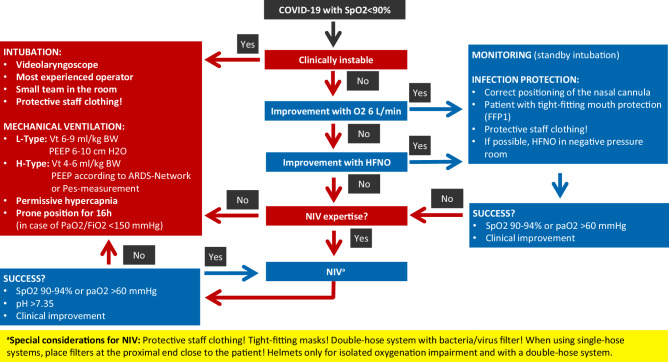
Guidance for the respiratory management of severe SARS-CoV‑2 CAP

Two phenotypes of COVID-19 lung disease are distinguished (Fig. 4): the L‑type (low elastance) is characterized by good compliance, a poor response to recruitment maneuvers and deterioration when excessively high positive end-expiratory pressure (PEEP) is used (>10 cm H_2_O). The frequent severe oxygenation impairment is primarily due to vasoplegia with an altered ventilation-perfusion ratio and microthrombotic events.

In the L‑type, O_2_/HFNO application, NIV or invasive ventilation with lower PEEP (6–10 cm H_2_O) and prone positioning are usually effective. Higher tidal volumes are well tolerated without lung injury (ventilator induced lung injury, VILI).

The H‑type (high elastance) is characterized by poor compliance (<40 ml/cm H_2_O), a higher shunt, increased right cardiac pressure and a better response to recruitment maneuvers, and basically represents full-blown severe ARDS. Mechanical ventilation with a relatively high PEEP (invasively mostly >15 cm H_2_O), but frequently low plateau pressures is useful. It can be assumed that COVID-19 ARDS patients also benefit significantly from prone positioning according to the ProSEVA protocol [62, 75]. Recruitment maneuvers (Lachmann maneuvers) can also be tried in patients with the H‑type [76].

A transition from the L‑type to the H‑type is possible and may be recognized early due to increased breathing effort (esophageal manometry, change in CVP, assessment of the work of breathing).

According to present experience and autopsy reports, euvolemia is recommended because overhydration disproportionately worsens the respiratory situation.

To date, there is no substantial evidence for the application of the aforementioned experimental COVID-19 therapies for patients in intensive care. Based on the principle primum nil nocere, the use of insufficiently validated and unapproved medications is only recommended in clinical trials, or in compassionate use programs. Moreover, potential side effects and possible interactions with standard intensive care medication have to be considered [77]. Equally, the evidence for efficacy of a supportive therapy with zinc, ascorbic acid or selenium is also insufficient.

The WHO guidelines for the treatment of COVID-19 incorporate the subject of intensive care and we recommend the regular updates to be followed and accounted for [62].

Microcirculatory disturbances on a thrombotic basis are assumed, and after a risk-benefit analysis a pharmacologic thrombosis prophylaxis is also indicated for the frequently occurring (moderate) thrombocytopenia [78, 79].

As occurs during other serious infections, COVID-19 ARDS patients may develop a form of secondary hemophagocytic lymphohistiocytosis (sHLH). Therefore, a close watch must be kept for signs of a massive hyperinflammatory response. Specific and adequately evaluated diagnostic criteria for COVID-19 sHLH are not yet available [80, 81]. Diagnosis and classification of sHLH so far have been based on the practice-oriented and evaluated HScore [82, 83]. A freely available calculator can be found at http://saintantoine.aphp.fr/score/. There is no gold standard for the therapy of sHLH; the current evidence is based on case series, and RCTs have yet to be conducted. As with other non-COVID-19 associated sHLH, in individual cases especially systemic corticosteroids, but also cyclosporine, intravenous immunoglobulins, anakinra, tocilizumab or other therapies may be considered [84].

#### Aerosol therapy

During any form of inhalation or respiratory support therapy (nebulization, O_2_ via nasal cannula/mask, HFNO, NIV), aerosol formation and thus an increased risk of infection for healthcare professionals and patients must be expected (see also section on “Cardiorespiratory physiotherapy”) [85]. These treatment forms should only be used if indicated, and in view of the possible risk of contamination of the surroundings by aerosols should either be applied in a relatively restrictive manner or even avoided. Preferably, bronchodilators or corticosteroids should be inhaled with dry powder inhalers or (also with NIV or invasive ventilation) metered dose inhalers [86].

For further details see the sections on “Respiratory intensive care” and “Cardiorespiratory physiotherapy”.

#### Hospitalized COVID-19 patients with sleep-related breathing disorders

If a patient treated with positive airway pressure (PAP) for a sleep-related breathing disorder develops COVID-19, it may be assumed that analogous to NIV and aerosol therapy, this therapy increases virus transmission to the environment. In this case, an individual risk-benefit assessment must be performed; however, if possible, PAP should be continued under strict hygiene and isolation measures. According to current evidence, PAP does not exacerbate COVID 19 infections. When single-hose systems and vented masks have been used so far, for the protection of the practitioner it is recommended to not use air humidifiers if possible and change to non-vented masks with a special exhalation valve and filter. If available, switching to a two-hose system is an alternative option.

#### Bronchoscopy in COVID-19 patients

Bronchoscopy is not recommended for the exclusion or verification of COVID-19 (lack of therapeutic consequence, unnecessary risk for personnel, and possible risk of clinical deterioration due to bronchoscopy); however, in exceptional situations, bronchoscopy may be indicated in confirmed or suspected COVID-19 patients (e.g. in immunosuppressed patients to exclude *Pneumocystis* pneumonia).

Bronchoscopy involves the risk of aerosol formation and thus a significantly increased risk of SARS-CoV‑2 infection for personnel present during the procedure. Bronchoscopy in intubated patients probably has a lower transmission risk.

In accordance with international recommendations, if SARS-CoV‑2 infection is suspected or confirmed, the following should be considered during the COVID-19 pandemic [87–89]:

- Extremely restrictive indications for a bronchoscopy.
- Primary use of other sensitive diagnostic procedures (e.g. obtaining tracheal secretions via a closed suction system for microbiological testing including SARS-CoV‑2 PCR).
- Bronchoscopy is indicated in emergency situations (e.g. life-threatening hemoptoe, high-grade airway stenosis, or foreign body aspiration), or if an alternative diagnosis can be verified, which would lead to a significant change in therapeutic management.
- Reduction of staff (bronchoscopist, bronchoscopy assistance, if necessary an anesthesia team) to a core team. No students, basic or advanced trainees in the bronchoscopy suite.
- Strict personal protection for the entire team (disposable protective gown, disposable gloves, FFP3 mask, protective glasses/visor, hair protection). Strict attention to correctly putting on and taking off protective clothing.
- If justifiable, rigid bronchoscopies with jet ventilation should not be performed; however, should a rigid bronchoscopy be unavoidable, it should be performed in an intubated patient with conventional ventilation and reduced aerosol escape, e.g. using a FLUVOG attachment (KARL STORZ SE & Co. KG, Tuttlingen, Germany).
- Bronchial lavage should be performed as fractionated procedure (10 ml NaCl 0.9% for each fraction; to reduce the transmission risk, the suction device should be clamped after sampling or before disconnection).
- Bronchoscopes are to be cleaned and disinfected in a validated manner; there is no evidence that these processes have to be changed for SARS-CoV‑2.

Routine bronchoscopies in non-COVID-19 patients (e.g. for the evaluation of pulmonary nodules/lesions or interstitial lung diseases) should only be performed during the current pandemic if strictly indicated, with increased personal protection measures (including the use of FFP2 or FFP3 masks) and strict adherence to hygiene protocols.

#### Therapeutic goals, treatment limitations and withdrawal of treatment in COVID-19 patients

**Fig. 5:**
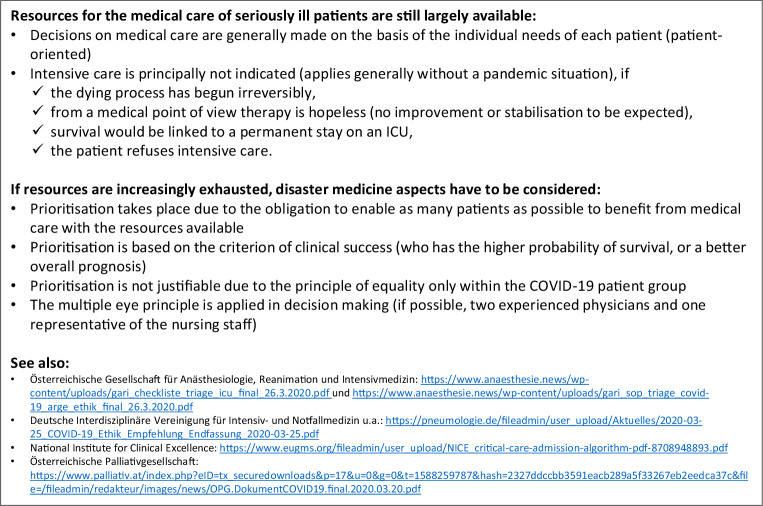
Guidance on limitations/withdrawal of therapy (DNE, DNI, DNR etc.) during the COVID-19 pandemic. *DNE* do not escalate, *DNI* do not intubate, *DNR* do not resuscitate

The ethical principles of intensive and palliative care apply equally to COVID-19 patients. Since in several countries even increased intensive care resources have been completely exhausted, guidelines for the allocation of intensive care beds, triage and palliative care have been established in Austria [90, 91]. Based on the patient’s present state of health and the severity of the infection and respect for the will of the patient, capacities should be kept available for patients for whom a higher probability of survival is predicted [92]. Not only is this a difficult undertaking due to the lack of validated predictive scores for COVID-19, but it also ignores the problem that patients without SARS-CoV‑2 infection, or those with clinically silent infection, may require intensive care for other reasons (e.g. COPD exacerbation, myocardial infarction, polytrauma, etc.) (Fig. 5). The German and British professional societies have developed recommendations regarding clinical-ethical decision-making [93, 94].

## General management of patients with chronic lung disease during the COVID-19 pandemic

### Measures for the prevention of COVID-19 and/or severe courses of the illness (recommendations for patients with underlying diseases)

**Fig. 6:**
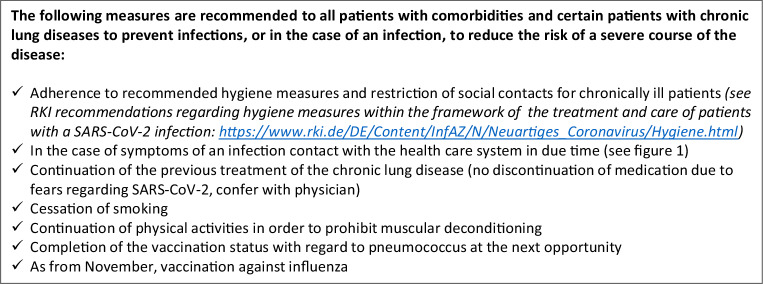
Patient information: preventive measures to prohibit COVID-19 and/or a severe course of the disease (recommendations for patients with underlying diseases)

Patients with chronic lung diseases may protect themselves from serious infections or, in the case of an infection, may reduce the risk of a severe course of the illness. The following measures are of particular importance (Fig. 6):

- Adherence to currently recommended hygiene measures and contact restrictions for chronically ill patients.
- Early contact with the healthcare system, if signs and symptoms of infection develop (Fig. 1).
- Continuation of the current therapy of the chronic lung disease (no discontinuation of medication for fear of SARS-CoV‑2, consultation with the attending physician).
- Immediate cessation of nicotine consumption, since smoking significantly increases the mortality risk of COVID-19 patients [95].
- Physical activity to prevent muscular deconditioning.
- Completion of the vaccination status with pneumococcal vaccine at the next opportunity.
- Influenza vaccination from November 2020.

In addition, during the COVID-19 pandemic it may be considered that especially vulnerable patient groups should be temporarily exempted from professional activities in public places. The aim of this measure would be to reduce the number of seriously ill people in order to relieve pressure on the healthcare system. Based on current data and clinical experience with other acute respiratory virus infections, this can be considered for the following groups of patients with chronic lung diseases:

- Age >65 years and a severe lung disease of any kind
- Age ≤65 years with
  - pO_2_ <65 mm Hg at room air or
  - long-term oxygen therapy (LTOT) or
  - FEV1 <70% of reference value or
  - FVC <70% of reference value or
  - diffusion capacity <70% of reference value or
  - cystic fibrosis or
  - active cancer or
  - systemic immunosuppressive therapy or congenital immunodeficiency.

Patients ≤65 years with only mild COPD or mild asthma and well-controlled symptoms have no (significantly) increased risk of developing severe COVID-19. Therefore, they do not require a general leave of absence from work.

#### Influenza vaccination

The 2020/2021 influenza season will begin in about half a year, when SARS-CoV‑2 will probably still be a burden to the healthcare system. In view of the known high annual influenza morbidity and mortality rate and the chronically low influenza vaccination coverage rate in Austria (general population <10%, and risk groups <25%), a double burden with COVID-19 and influenza has to be expected as of December 2020. Consequently, we recommend that the required effective prevention measures for the 2020/2021 influenza season are initiated immediately. In the opinion of the Austrian Society of Pneumology and the Influenza Task Force Austria, these should include the offer of the influenza vaccination in Austria at a much lower threshold and free of charge (as is common in most European countries).

### Asthma and COPD

#### Asthma

In patients with well-controlled asthma, in general there is no increased risk of a more complicated course of COVID-19; however, the situation is different when asthma is poorly controlled. Therefore, it is of particular importance that asthma patients are very careful in taking their medication as this can contribute to good illness control. This applies in particular to inhaled corticosteroids, which help to control this chronic inflammatory disease. Treatment with biologicals (such as omalizumab, mepolizumab, reslizumab, benralizumab, and dupilumab) should also remain unchanged. According to present knowledge a negative influence on the immune defence against SARS-CoV‑2 is not expected from these biologicals, but from oral corticosteroids (the therapeutic alternative).

Under no circumstances should the drugs be discontinued on the assumption that they could impair the immune system; a well-controlled asthma is the best provision for a mild course of a SARS-CoV‑2 infection. In addition, patients with asthma should pay close attention to any marked changes in their symptoms, especially to a sudden increase in breathlessness and newly occurring cough and fever. While shortness of breath and cough are common in patients with asthma, fever may possibly indicate an infection and should be taken seriously and further assessed.

#### COPD

COPD patients should also adhere strictly to their regular therapy in order to prevent exacerbations. If in the current situation an exacerbation occurs and requires medical consultation or hospitalization (Fig. 1), patients cannot follow the most important recommendations for the prevention of a SARS-CoV‑2 infection, namely staying at home and keeping at a distance to other people. In general, regularly applied medication contributes to good disease control and this also applies to COPD; thus, a high degree of adherence to therapy is of advantage especially in this pandemic. Dyspnea and cough are typical COPD signs and symptoms; a sudden worsening of dyspnea and increased body temperature should prompt suspicion of SARS-CoV‑2 infection in these patients (Fig. 1); however, fever could also be due to a COPD exacerbation. While systemic corticosteroids are currently not recommended for COVID-19, their use for the treatment of a common COPD exacerbation is justified.

#### Asthma and COPD patients with probable or confirmed SARS-CoV-2 infection

Patients with chronic respiratory diseases and a SARS-CoV‑2 infection have an equal chance of a mild course of the disease that can be treated in domestic isolation. High grade fever should be avoided, and sufficient hydration is recommended. Even in stable phases of their disease, many COPD patients control oxygen saturation independently with finger pulse oximetry. If the oxygen saturation falls below the usual range, medical care should be sought. If it is not possible to control oxygen saturation at home, breathing should be closely monitored. If dyspnea at rest or during minimal physical activity increases, medical care should be sought. Asthma patients should document their symptoms and peak flow values in the usual manner. Marked changes require medical attention. Generally speaking, COVID-19 can temporarily worsen the respiratory symptoms of patients with chronic lung diseases, but the patients usually recover without consequences, including a complete recovery of lung function.

### Lung cancer

There is currently no evidence to suggest that discontinuing or interrupting anti-tumor therapy, such as chemotherapy and/or immunotherapy is necessary. Diagnosis and therapy should be continued according to current standards; however, an individual decision should always be reached between the physician and the patient. For further details we suggest consulting the current ASCO, ESMO and DGHO/ÖGHO recommendations [96–98].

### Cystic fibrosis (CF)

Adult CF patients are at risk of a possibly severe disease course with SARS-CoV‑2 infection. Apart from adherence to the generally valid prevention measures (hand hygiene, social distancing) adult CF patients should stay at home and not seek social contacts in professional or other types of social environment. Third parties should undertake shopping for food and supplies of medications or respiratory physiotherapy devices and the purchases should be placed in front of the door.

Furthermore, with respect to routine appointments in outpatient clinics, contact should be made with the respective center in order to clarify which examinations can be postponed, and which outpatient visits might take place under special conditions in the respective unit, or whether in individual cases the visit can be replaced by telephone consultations and instructions.

The CF centers are making every effort to suspend outpatient visits that are not absolutely necessary as long as this does not cause disadvantages for the patients. The usual therapeutic measures such as chest physiotherapy, medical, and nutritional therapy should be continued in a particularly careful manner. In the case of clinical deterioration, signified by fever and increased cough with or without respiratory distress, it is advisable to contact the responsible CF centre by telephone, especially if a visit to the center has already been scheduled.

Clearly indicated inpatient i.v. antibiotic treatment courses should be administered in any case. If inpatient treatment of a SARS-CoV‑2 infection is necessary, a tailored antibiotic therapy adapted to the respective microbial spectrum will be initiated.

A recently published article reported on ten SARS-CoV‑2 infected CF patients in Lombardy (out of a total of 42,161 infected people in Lombardy and 101,739 in Italy on 31 March 2020). In each case, the infection had been transmitted by a family member. In addition, five patients were reported from France, seven from the UK, five from Germany and three (including one transplantation patient) from Spain (all of them adults) [99]. In this limited number of patients, the SARS-CoV‑2 infection did not lead to a noticeable worsening of the underlying disease. The CF centers are encouraged to report patients infected with SARS-CoV‑2 to the European CF registry (servicedesk@ecfregistry.eu).

### Interstitial lung diseases

Due to structural lung changes, immunosuppressive therapy, diffusion impairment with a frequently existing need for supplemental oxygen and advanced age, patients with interstitial lung disease (ILD) are a COVID-19 risk group. In order to minimize the risk of infection, ILD patients should adhere rigorously to social distancing and other recommended protective measures. Support from family members, neighbors and aid organizations with respect to the organization of supplies of food and medication is essential, although at the same time direct contact with people not living in the same household should be strictly avoided.

Scheduling of appointments in ILD outpatient clinics should be optimized in order to avoid long waiting times and patient crowding. With written consent and by means of technical support, alternatives such as video chats can be considered for routine follow-ups. To minimize direct contact between physicians and thus the risk of infection transmission, alternative (e.g. digital) forms of communication should also be considered for multidisciplinary case discussions (ILD boards).

For a timely diagnosis of a SARS-CoV‑2 infection, it is necessary to perform PCR testing as soon as new signs and/or symptoms of illness develop. This allows the early detection of other causes of the symptoms or an acute exacerbation and appropriate treatment can be initiated without delay.

Many ILD patients are treated with immunosuppressive agents. Thus, in the case of a viral infection more severe disease courses can be expected. Antifibrotic therapy in fibrosing ILD and immunosuppressive therapy in inflammatory ILD should be continued in ILD patients, who are not suffering from COVID-19, in order not to risk ILD exacerbation. If a SARS-CoV‑2 infection is confirmed, an individual assessment must be made as to whether immunosuppressive therapy should be reduced or temporarily discontinued.

Treatment of patients with advanced ILD and COVID-19 is likely to generate ethical concerns and difficult therapeutic decisions may be required. An open discussion of the issues with patients and their families and the definition of treatment goals may be necessary. For patients with advanced ILD and COVID-19, palliative measures should also be considered.

### Pulmonary hypertension

Patients with pulmonary hypertension, and in particular pulmonary arterial hypertension (PAH), belong to the risk patient group; however, there are no data on the clinical course of COVID-19 in patients with PAH. We are also unaware of any recent publications that have investigated specific correlations between this viral disease and pulmonary vascular disease.

As with other lung diseases, infection prevention is of general importance in patients with PAH. Depending on the severity of the underlying disease, even mild respiratory infections have been reported to cause a temporary increase in the pressure load of the right heart up to clinical decompensation. A pneumonia caused by SARS-CoV‑2 leads to a deterioration of oxygenation, and the accompanying local and systemic inflammatory reactions also suggest the possibility of a worsening of the right ventricular function. In an autopsy study, an accumulation of marked right ventricular dilatation in deceased COVID-19 patients was described [100].

As a consequence, the officially recommended measures for social distancing appear to be of significant importance for patients with pulmonary vascular diseases; however, this should not result in delayed diagnostics. Suspected cases of acute pulmonary embolism should continue to be assessed and treated according to guidelines as soon as possible in order that patients with a potentially fatal acute illness are not harmed. Patients with suspected severe pulmonary hypertension should also be subjected to examinations including right heart catheterization without delay and treatment should be initiated in accordance with the guidelines.

Patients with PAH therapy should adhere to the generally recommended hygiene and other measures and, if there are signs and/or symptoms of a SARS-CoV‑2 infection, depending on the severity of the symptoms, they should contact their general practitioner, consultant or specialist at the centre and start antibiotic therapy early.

The need for a regular outpatient visit at the PAH centre should be assessed on an individual basis. Patients should take precautionary measures with regard to their specific PAH medication (supplies for at least 8 weeks) and, if necessary, in the case of supply shortages duly contact the PAH centre. Close telephone contacts with patients are recommended and should be practiced by the centers.

### Pulmonary rehabilitation and smoking cessation therapy

The Pension Insurance Fund (*Pensionsversicherungsanstalt*, PVA) is classified as being part of the critical infrastructure of Austria. It is legally obliged to maintain services and in particular those of its own rehabilitation centers. In the health service area, the inpatient rehabilitation centers continue to provide care and rehabilitation to those who urgently in need following acute medical events or interventions. At the same time, all non-urgent measures are currently reduced [101].

The pulmonary outpatient and inpatient rehabilitation centers are closed until further notice due to the risk profile of the patients affected. Spatial and personal structures were organized in cooperation with surrounding hospitals in order to provide adequate patient care.

At the end of the COVID-19 pandemic the rehabilitation centers expect a great demand for rehabilitation treatment due to the backlog caused by the postponement of rehabilitation treatment and the high incidence of COVID-19 victims. This anticipated demand should be considered in advance.

### Sleep-associated breathing disorders

In line with the current statement of the German Society for Sleep Medicine [102], we agree with the following assessment: there is no reliable information on whether sleep apnea patients have an increased risk for SARS-CoV‑2 infection, or are subject to a greater risk of a severe course of the disease; however, many sleep apnea patients are older people (>65 years) and in addition suffer from typical concomitant or secondary sleep apnea diseases (lung, heart, kidney, liver diseases, severe obesity, diabetes mellitus), which are considered risk factors for a severe course of COVID-19. Apart from the general recommendations of the health authorities, no evidence-based specific recommendations for asymptomatic patients with PAP therapy can be made at present. For cleaning and disinfection of CPAP devices, the specifications of the respective manufacturer are pivotal.

Due to the possible environmental SARS-CoV‑2 exposure via CPAP/APAP, a temporary separation of bedrooms during the pandemic can be discussed with concerned asymptomatic patients or relatives [103].

### Transplantation

To date, details of very few patients with COVID-19 following solid organ transplantation have been published; however, the issue is being observed and discussed within transplant networks [104]. Similar to the general population, SARS-CoV‑2 infection after transplantation can vary widely. A greatly increased risk of a severe course of COVID-19 has yet to be identified amongst transplant patients [105].

In immunocompromized patients, symptoms may start atypically with gastrointestinal symptoms and fever, while pulmonary symptoms occur at a later point [106]. Thus, COVID-19 should also be considered in transplant patients with extrapulmonary symptoms.

Most transplantation programs were temporarily suspended during the COVID-19 pandemic. Only high urgency cases should be transplanted at present and all donors and recipients are being tested for SARS-CoV‑2. The standard follow-up after transplantation will be temporarily minimized and in particular routine hospital visits should be reduced; if applicable, more use should be made of telemedical methods (e.g. www.daag.de).

The management of transplant patients with a SARS-CoV‑2 infection is not yet standardized. In asymptomatic patients no change in therapy should be made. If symptoms arise, a break with respect to mycophenolate or azathioprine is recommended, and the dosage of calcineurin inhibitors should be reduced. The Austrian Society of Nephrology recommends a similar procedure after kidney transplantation [107]. Whether or not a dose increase of corticosteroids in lung transplant patients is advisable cannot yet be answered.

Those COVID-19 ARDS patients with refractory respiratory mono-organ failure (unsuccessful weaning) may be considered for lung transplantation once the florid infection has subsided, depending on their age, general condition and the absence of relevant comorbidities [108].

### Cardiorespiratory physiotherapy

Like most viral pneumonias, SARS-CoV‑2 CAP is an interstitial pneumonia. Accordingly, SARS-CoV‑2 infection does not produce relevant purulent secretion in the intra-alveolar and bronchial airspaces and a dry, non-productive cough is a major symptom. In these cases, chest physiotherapy is unnecessary; however, exudative consolidations (e.g. due to secondary bacterial infections) and hypersecretion with difficulties in removing secretions may occur in the course of COVID-19, or if certain underlying diseases are present. This not only applies to patients with underlying severe obstructive pulmonary disease (COPD, asthma), CF, or non-CF bronchiectasis, but also to patients with neuromuscular diseases or spinal cord injuries. In such situations, chest physiotherapy may be indicated in the presence of current or foreseeable problems with the removal of secretions. Individual assessments must be made for each patient in order to ascertain if the intervention justifies the possible risk of staff infection (no unnecessary therapies).

#### Physiotherapeutic interventions with a potentially high virus exposure

These include:

- Inhalation training, secretion-removing techniques and sputum induction
- Manual and mechanical cough assist
- Oxygen therapy (also with nasal cannula)
- Intermittent positive pressure breathing (IPPB) and NIV
- Airway suctioning
- Mobilization and training
- Care for tracheotomised patients
- Inspiratory and expiratory muscle training

Any unnecessary contact (e.g. common routine visits) should currently be avoided in cases of confirmed or suspected SARS-CoV‑2 infections and, if possible, assessments should not be carried out with direct physical contact (possibly telephone contact, transfer of information via nursing staff).

All physiotherapeutic techniques that potentially stimulate coughing or mobilize secretions increase the risk of virus transmission. This risk must be weighed up carefully in each individual case and appropriate safety measures must be taken.

#### Aerosol therapy

Aerosol therapy in non-intubated patients with COVID-19 using jet/membrane or ultrasound nebulizers is not recommended, as it can lead to increased virus release into the ambient air similar to NIV, HFNO or pulmonary function testing. Instead, aerosol therapy with a metered dose inhaler in combination with a spacer is preferred. If nebulization is inevitable, a virus filter should be inserted before the expiration valve and appropriate protective measures for medical staff taken.

#### General rules for physiotherapeutic interventions in intubated/tracheotomized patients with COVID-19 or suspected COVID-19

- Suctioning in intubated/tracheotomized patients should only be performed with a closed suction system.
- Disconnection of ventilated patients from the ventilator should generally be avoided; if absolutely necessary it should be carried out with a clamped tube and deactivated ventilator.
- Deflation of the cuff of a tracheal cannula and cleaning of the inner cannula potentially create the risk of airborne virus transmission.
- To minimize the risk of virus transmission, inspiratory muscle training and the use of speaking valves with tracheal cannulas should only be carried out after the acute infection has subsided.
- Obligatory use of personal protection equipment (mask, goggles, long-sleeved protective coats; gloves; staff with beards should remove facial hair to enable a proper fit of the face mask; for interventions that increase the airborne virus load, hair protection has to be worn).

#### General additional recommendations for chest physiotherapy in non-intubated patients

- Obligatory use of personal protection equipment (see above)
- Adherence to cough etiquette (applies to staff and patients):
  - During coughing and expectoration avert the head.
  - Secure the secretions in a tissue or a container and dispose of it immediately; subsequent obligatory hand disinfection.
  - For intentional coughing maneuvers: minimum distance of 2 m and/or moving out of the likely path of dispersion.
- Physiotherapeutic interventions only on request/after consultation with the responsible physician.
- In case of an essential physiotherapeutic intervention with a potential risk of virus transmission:
  - Completion in a single-bed room with the doors closed.
  - Minimum staffing with all those present wearing personal protective equipment.
  - Use of one-way products.
- No sputum induction.
- No manual hyperinflation, if a mechanical option is available.

In general, the same safety precautions apply to physiotherapeutic interventions concerning mobilization, training and rehabilitation. Therapeutic equipment must be disinfected or disposed of after use and personal safety measures must be followed in accordance with local regulations.

This short summary of the most important measures is based on a recent article [109].

### Respiratory nursing

The care for COVID-19 patients or people with suspected COVID-19 constitutes a major challenge for nursing staff. The need for professional nursing and care varies depending on the course of the illness and limitations due to pre-existing comorbidities.

For nursing staff, the early detection of potentially transmissible and life-threatening infections and strict adherence to protective measures during care are of immanent importance. These protective measures not only serve as personal protection for healthcare professionals, but also help prevent nosocomial infections.

The protection measures comprise [110–112]:

- Consistent adherence to personal hygiene measures:
  - Frequent hand washing
  - The avoidance of contact with eyes, nose and mouth
  - Sneezing and coughing preferably into a handkerchief, which should immediately be disposed of
  - A minimum distance of 2 m to other people
  - Hand hygiene in all health care areas
- Organizational precautionary measures:
  - Reduction and control of patient flow in order to prevent transmission from patient to patient.
  - Provision of infected patients with mouth and nose protection if the patient’s state of health permits.
  - Spatial distancing of suspected patients from other people, ideally in an isolation room with a vacuum lock and its own hygiene facilities.
  - Reduction of patient transport to an absolute minimum and the provision of the transport staff with advance information.
  - Reduction of social contacts (visitors in the hospital) by an increase in telecommunication, limitations of the number of visitors and the duration of each visit, as well as instruction of visitors regarding hygiene measures.
  - Information, education and instruction of the relevant staff on protective measures and monitoring their own health status.
  - Reduction of inward and outward transfers for the treatment and care of infected patients by cohorting them under certain circumstances and planning and merging their care measures.
  - Disinfection and cleaning of patient-near, contaminated, or probably contaminated surfaces and used medical devices with disinfectants possessing at least limited viricidal effectiveness.
- Personal safety measures/personal protective equipment:
  - The choice of appropriate personal protective equipment depends on the nature and extent of patient care.
  - Due to the current worldwide demand for personal protective equipment, the available products should only be used in a resource-protective manner (reuse in compliance with the respective manufacturer’s specifications, ensuring correct interim storage).
  - Putting on and taking off personal protective equipment should be practiced regularly; for COVID-19 patients only trained staff should be deployed.
  - In completely isolated areas (e.g. an entire hospital ward) it should be ensured that the shifts of nursing staff, who constantly wear protective equipment, do not exceed 3–4 h before a break can be taken, in order to prevent pressure-related injuries from the protective equipment. Hydrocolloids have proved to be effective for skin protection.
  - Hand disinfection using disinfectant agents with at least limited viricidal capacity before protective equipment is put on, gloves are removed, and prior to leaving the room, as well as in accordance with other standard hand hygiene procedures.
  - For care measures involving aerosol production, masks should be worn that stop at least 95% of all particles with a diameter of >0.3 µm. This corresponds to the FFP2 class of the respiratory mask classification system used in Europe.
  - The minimum personal protective equipment for the direct care of COVID-19 patients, or patients suspected of being SARS-CoV‑2 positive, consists of FFP2 masks, safety goggles or a face shield, a long-sleeved water-repellent protective gown and disposable gloves.

The measures implemented to contain the infection have the support of the majority of the general public. This and the variable course of COVID-19 cause anxiety in those affected, which can be further increased by isolation and protection measures. The reduction or ban of visits to hospitals, nursing homes and homes for the aged are conducive factors to anxiety, loneliness and social isolation. The dilemma created by isolation as a protective measure and its social, psychological and physical consequences has been the subject of numerous studies and has also been described by the German Ethics Council as a major ethical conflict in the current situation. Consequently, apart from relationship work, the support from caregivers in daily life activities represents an essential measure for the avoidance of negative consequences for affected patients [113].

Measures to cope with the anxiety of affected and isolated patients mainly consist of methods of communication; however, these are impeded by the physical distance rule, spatial distancing and the wearing of personal protective equipment. Above all, palliative situations, the dying process and the support of relatives, who have lost someone and wish to bid farewell, are exceptional challenges for health care professionals. Furthermore, for persons with dementia, delirium or other mental illnesses, isolation is also difficult to accept. There are approaches to stabilize the home care of people suffering from dementia. Moreover, recommendations regarding the documentation of the dying process are available [114–117].

### Pulmonary function testing

In the critical phase of the COVID-19 pandemic, outpatient clinics and pulmonary function laboratories should take appropriate measures to avoid crowding (for example in the waiting area). Furthermore, at present increased efforts are required for disinfection and cleaning after each pulmonary function testing. Therefore, the need for special examinations (e.g. cardiopulmonary exercise testing) should be critically evaluated. Before each examination, patients should be asked about signs and symptoms of infection in a standardized manner. In case of doubt, pulmonary function testing should not be carried out or postponed.

Patients without any signs or symptoms of infection can still be examined, although during the pandemic the staff present in the room during the examination should wear mouth and nose protection (FFP2 or FFP3 mask). Patients who have recovered from a SARS-CoV‑2 infection (no evidence of prolonged infection, no symptoms, PCR 2 × negative) can also be examined. Pulmonary function laboratories should also have a stock of extended protective equipment (safety goggles, protective gowns).
